# Live Streaming of the Professor’s Ward Rounds in Undergraduate Neurology Education: Usability Study

**DOI:** 10.2196/50128

**Published:** 2024-08-22

**Authors:** Kenji Sekiguchi, Seiji Kawano, Norio Chihara, Seimi Satomi-Kobayashi, Eiichi Maeda, Riki Matsumoto

**Affiliations:** 1 Division of Neurology Kobe University Graduate School of Medicine Kobe Japan; 2 Integrated Clinical Education Center Kobe University Hospital Kobe Japan; 3 Division of Medical Informatics Kobe University Hospital Kobe Japan

**Keywords:** live streaming, ward round, web conferencing software, neurological examination, undergraduate education, medical student, medical education, rounds, remote education, video-conferencing, residents, live-stream, neurology, neuroscience, web conferencing, distance education, technology enhanced learning, mobile phone

## Abstract

**Background:**

Although neurology department ward rounds are among the most important medical education exercises in Japan, they have several issues. Patients may find it unpleasant to undergo repeated neurological tests, especially when in the presence of several students. Only the front row of students can closely observe the examination findings; moreover, students were prohibited from contacting patients altogether during the COVID-19 pandemic. One possible solution is to use commercial videoconferencing systems. However, Japanese patients are reluctant to have their medical information or video footage of them sent outside of the hospital via the internet.

**Objective:**

The study aimed to confirm the feasibility of conducting remote teaching rounds using an in-house web conferencing system in which the patients’ personal data are securely protected. This study also explored whether using remote rounds alongside face-to-face participation would enhance learning.

**Methods:**

We created an on-premises videoconferencing system using an open-source app. To perform video ward rounds, the professor wore a wireless microphone while leading routine in-person rounds and the attending physician carried a tablet device linked to a web conference, allowing students in another room to watch the rounds on a live stream. In total, 112 of 5th-year students who entered their 1-week neurology rotation between 2021 and 2022 were instructed to participate in 1-hour in-person and remote rounds. Students were given questionnaires to evaluate their satisfaction and the educational effects of the remote rounds.

**Results:**

The remote ward rounds were conducted easily with no interference with the in-person rounds, nor any complaints from the patients. Each examination technique was explained by another teacher to the students who participated in remote rounds in the conference room. Characteristic neurological findings, such as plantar reflexes (Babinski sign), which are usually seen only by close observers during in-person rounds, could be visualized under magnification by all students. The postexperience survey (82/112, 73% response rate) showed that the mean score of participants’ satisfaction was 3.94 (SD 0.83; excellent 5 and poor 1). No participant scoring 1 was noted. The proportion of students who observed 6 representative abnormal neurological findings (Babinski sign, hyperreflexia, cerebellar ataxia, involuntary movement, muscular weakness, and abnormality in sensory examination) increased by 22% (18/82, range 13-24) compared to in-person rounds alone. When self-rating the learning value, 43% (35/82) of the students answered that remote rounds are equally as valuable as in-person rounds, while 32% (26/82) preferred remote rounds.

**Conclusions:**

Live-streaming of neurology ward rounds using a secure in-house web conferencing system provides additional learning experience without concerns regarding leakage of patient information. This initiative could enhance neurology learning before entering a clinical clerkship.

## Introduction

Ward rounds are considered one of the most important tools of clinical neurology education in Japan [1]. A professor or clinical tutor performs a neurological examination of the patient and describes the findings for educational purposes in the presence of a small group of 6 to 7 undergraduate students. The students are shown the focus areas for the neurological examination procedure, the order of performance, findings that are specific to the patient and not to the disease, and nontechnical skills such as manners and attitudes toward patients and methods of explaining and encouraging patients, thereby fostering professionalism [2-4]. These rounds afford students, who have only learned about normal findings in the Objective Structured Clinical Examination, their first opportunity to observe abnormal neurological findings and encourage their interest in neurology and further education. One of the most important learning objectives of the 5th-year neurology clinical rotation is to confirm one’s diagnosis by attending the professor’s rounds after having previously examined the assigned patients and attempted to localize their lesions [1].

While beneficial, from the patient’s perspective, it is not pleasant to be put on display in the presence of several students who have not signed a medical contract and endure numerous repetitive tests, despite experiencing painful symptoms. Only a few students can observe the examination up close since the postgraduate residents occupy the best places, and some students must watch small muscle twitches and pupillary changes in patients from the second and third rows, where it is also difficult to hear the professor’s explanation. This ceremonial event is therefore simplified in some universities, and the opportunity to observe abnormal neurological findings may not be available until the final-year elective clinical clerkship, when the students have close contact with patients [1].

Early in the COVID-19 pandemic, medical students were prohibited from being directly involved in patient care, and teaching rounds were almost completely canceled in Japan. During this time, students had no access to patients and were not allowed to enter the hospital and observe medical procedures [5].

The application of web-based conference apps such as Zoom (Zoom Video Communications) or Teams (Microsoft Corp), became rampant and changed the structure of society, although their use was limited in the medical field, where personal information cannot easily be divulged in the online space. These third-party servers exist outside of the hospital and in some cases, are operated from abroad. The Japanese public was reluctant to place their medical data and personal information onto these systems, even temporarily. Therefore, it is not possible to easily share images or videos of the patient (on a screen) without substantial investment in a system that is structured in compliance with various regulations devised by authorities to ensure secure handling of the patient’s protected information [6]. Medical students have not been able to benefit from technological advances because universities are hesitant to invest in new educational systems, since there is no direct revenue to be gained.

WebRTC (Web Real-Time Communication) is a technology used for real-time exchange of audio, video, and other data between web browsers. It constitutes the core technology for several commercially available web conferencing apps. We built our own educational web conferencing app system in the hospital using BigBlueButton, an open-source conferencing app based on WebRTC. The system allows the sharing of images within a closed network in the hospital, without using the internet, which is more acceptable to patients. Using this system, we attempted a clinical practice in which the professor’s weekly rounds, which are important in clinical education during neurology rotations, are live-streamed to medical students sitting in a separate room while another teacher provides explanations of the procedures. We examined the feasibility of remote ward rounds using this system and its effectiveness compared to the conventional in-person ward rounds.

## Methods

### Establishment of an On-Premises Secure Videoconferencing System in a University Hospital

In Japan, hospital information systems must conform to the Guidelines for the Safe Management of Medical Information Systems (version 6.0), established by the Ministry of Health, Labour and Welfare [6]. This law states that when patient information is moved from the hospital, including via the internet, various security risk management measures must be undertaken. These include information processing to prevent personal data leakage or using private or leased lines to reduce the possibility of information leaks en route. Japan does not have an authorization system such as the HIPAA (Health Insurance Portability and Accountability Act) in the United States, which imposes fines for violations; thus, medical information is generally not taken out of the hospital unless there is a specific reason. It is necessary to anonymize personal information and create generalized content when patient information is to be used for online medical education, rendering real-time clinical education difficult. We have created a web conferencing system called the KUMEX (Kobe University School of Medicine Web Conference Lecture System) that can only be operated within the hospital and is not connected to the hospital information system network, using BigBlueButton, an open-source conferencing app based on WebRTC. One of the features of KUMEX is that all communicating terminals are connected using encrypted point-to-point communication via a web browser, which establishes the connection over a VLAN (virtual local area network) within the university and not through a wide area network, to avoid the risk of information leaks enroute. Moreover, communication using the user datagram protocol with minimal delay is faster than the conventional client-server model. A signaling server is set up in the hospital to recognize each terminal, and audio and video media, from a webcam, can be shared and displayed on each terminal’s screen immediately. There is no need to install special apps, and anyone can participate in a meeting from any terminal on the same network by sharing a unique meeting code generated for each session. The app is installed on a server in the Division of Medical Informatics of Kobe University Hospital and can be used freely 24 hours a day. KUMEX meetings can be held on any terminal running a modern browser, whether a PC, tablet, or smartphone. No additional installation or operating costs were incurred.

### Preparation for Video-Streaming of Patients’ Examinations

Before the COVID-19 pandemic, inpatients were informed in advance about rounds that would be conducted by professors, residents, and students as an educational exercise involving another neurological examination to demonstrate abnormal findings. Patients were reassured that their privacy would be considered, and their verbal consent was obtained. When the educational rounds were video-streamed, we additionally obtained written informed consent stating that the examination would be streamed to the students located in another room of the hospital using KUMEX, that the connection point would be in the hospital and not via the internet, and that the video would not be saved without prior consent. Written declarations were obtained from the students to reconfirm their consideration for the handling of personal information when viewing and using the distributed images. Almost all patients who were asked to cooperate in this trial provided consent for participation.

### Video Ward Round Sharing to Undergraduate Students

The Kobe University curriculum requires a batch of 6 to 7 fifth-year medical students to attend clinics at the Division of Neurology on a rotating schedule every alternate week. Before the pandemic, the inpatient medical records were reviewed by all neurology staff and students in a conference room, following which all staff and students (more than 20 people) participated in in-person ward rounds with all inpatients. After the pandemic, only the attending physician, students assigned to the patient, and professor (approximately 5 people) visited the bedside for educational rounds, to reduce crowding and infection risk. To perform video ward rounds, the professor wore a wireless Bluetooth microphone (SmartMic+, Sabinetek) on the lapel of their white coat, while the attending physician carried a tablet device (iPad Pro 11-inch, Apple Inc). The attending physician filmed the examination at an easy-to-view angle. Residents and students not assigned to the patient remained in the conference room watching the professor’s consultation projected onto an enlarged PC screen used for KUMEX conferences. The remaining teaching staff described the neurological examinations performed by the professor, the medical terminology used to denote abnormal findings and their significance, and answered student questions. Communication was not 2-way and the iPad in the examination room did not carry audio from the conference room. Each student experienced 1 in-person rounds session and several remote rounds during this program. In total, 112 fifth-year students who entered their 1-week neurology rotation between 2021 and 2022 were instructed to participate in 1-hour remote and in-person rounds.

### Questionnaire Survey of Participating Students

As a method to determine student impressions and achievement, we chose a questionnaire that was easy to administer and less coercive. Some items were allowed multiple-response and some were allowed to be free-text. A web-based questionnaire was sent to fifth- and sixth-year students who attended the remote ward rounds within the year of this study. The contents of the questions are listed in Table 1. The survey included questions on satisfaction, advantages and drawbacks, self-rated learning value, and specific ratings regarding the opportunity to observe abnormal neurological findings, modeling professionalism, and improving motivation. It was not compulsory to answer every question. *z* tests were used to analyze whether there were significant differences in the proportions of each respondent (Q4-Q12). All the analyses were performed using GraphPad Prism software (version 8.0, GraphPad Software Inc).

**Table 1:** Postexperience survey of live-streamed remote neurology ward rounds.

Question number	Question	Response options	Implication
Q1	How did you feel about “remote participation in professors' rounds”?	Likert scale: 1 (poor) to 5 (excellent)	Satisfaction
Q2	What do you think are the advantages of participating in video rounds?	Select the prepared option or describe free text I could participate while seated I could closely observe the examination I could listen to the faculty’s commentary It was easy to understand because the points to focus on were shown Nothing Allow free description	Advantages
Q3	What do you think are the drawbacks of participating in video rounds?	Select the prepared option or describe free text Lack of an immersive experience Poor video and audio quality Lack of a sense of tension Nothing Allow free description	Drawbacks
Q4	What do you think of the learning effect of remote participation in professor’s rounds compared to following the standard rounds, if the learning time is the same?	Video ward rounds are better In-person rounds are better Both are equal	Self-rated learning value
Q5-Q10	Were you able to see any of the following abnormal neurological findings during either of your rounds during your neurology rotation? Babinski sign (extensor plantar response) Hyperreflexia (exaggerated tendon jerk response) Cerebellar ataxia on finger-to-nose test Involuntary movement (eg, tremor) Muscular weakness Abnormal sensory examination	Could observe in video ward round Could observe in both Could observe in in-person round Could not observe in either	Observation of the abnormal neurological finding
Q11	In which learning activity did you feel that the professors and supervisors were more attentive and caring toward the patients?	Felt in video ward round Felt in both Felt in in-person round Could not feel in either	Modeling professionalism
Q12	Did your neurology rotational training, including remote participation in professor’s rounds, influence your motivation during your subsequent clinical clerkship?	Influenced Unchanged	Improved motivation

### Ethical Considerations

All patients who participated in video-streamed ward rounds using KUMEX were informed and wrote consent forms approved by Kobe University Hospital as a clinical initiative in advance. Written declarations were obtained from the students who participated in this study to reconfirm their consideration for the handling of personal information when viewing and using the distributed images via KUMEX. The Ethics Committee of Kobe University Hospital does not review research related to education, as it is not subject to ethical review, and issued a certificate of exemption [7]. Therefore, we took the utmost consideration for the students and patients to ensure that the project could be carried out ethically. When answering the questionnaire, the significance of the research was explained to be understood by the students, and the opportunity to opt out was offered. Participants including patients and students did not receive any kind of compensation. All study data presented in this paper are deidentified.

## Results

### Video Streaming of the Neurology Ward Rounds Was Easily Enabled by KUMEX

**Figure 1:**
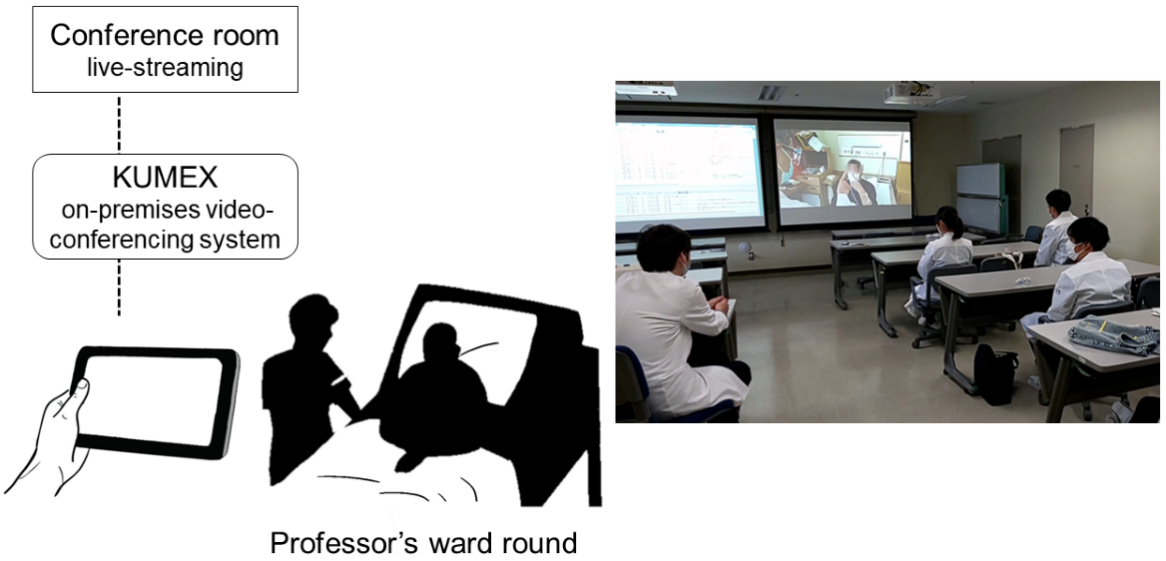
The schema of the system of a remote ward round using KUMEX which was established securely within the hospital network. Students watched the live-streaming of the professor examining an in-patient, projected onto a large screen in the conference room via KUMEX. KUMEX: Kobe University School of Medicine Web Conference Lecture System.

The students watched a real-time video streaming of the professor examining an inpatient, projected onto a large screen in the conference room via KUMEX (Figure 1). The communication was conducted over a VLAN within the university, distinct from the hospital information system, and both the audio and video were transmitted from the iPad via Wireless Fidelity with an insignificant amount of delay. The patients behaved naturally without being excessively conscious of being recorded. Characteristic findings, such as plantar reflexes (Babinski sign) and fasciculations of the tongue, which are usually only observed by the students in the front rows during in-person rounds, could be seen under magnification by all students. Each examination technique was explained by one of the teachers and questions from the students were answered briefly. These additional devices (the professor’s microphone and the iPad held by the attending physician) never interfered with the ward round stream and no complaints were received from the patients afterward.

### Results of the Student Questionnaire

**Figure 2:**
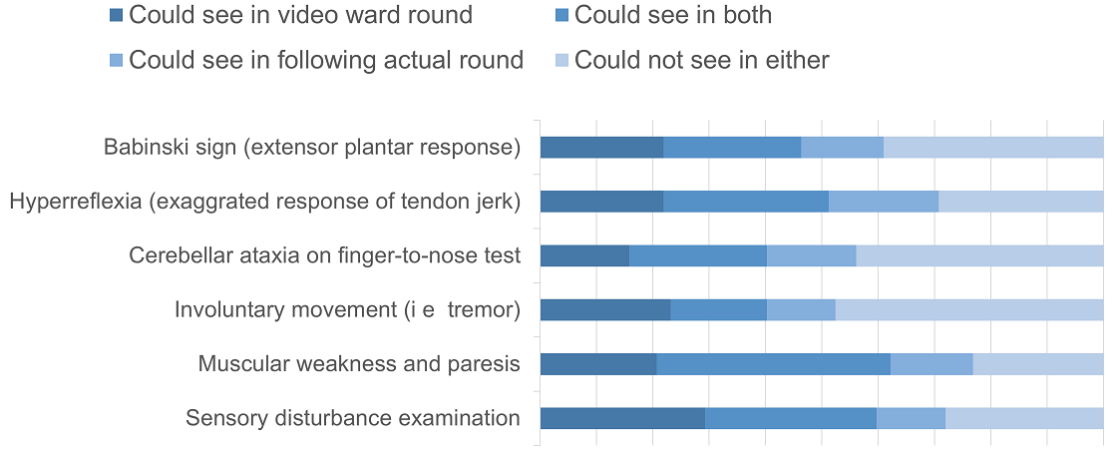
Percentage of students experiencing abnormal neurological findings during remote ward rounds, in-person rounds, and both (questions 5-10). Each student experienced 1 in-person rounds session and several remote rounds performed by the professor. The proportion of students who observed 6 representative abnormal neurological findings increased by 22% (18/82, 13-24) compared to in-person rounds alone.

Results of the postexperience survey for the students are shown in Tables 2 and 3, and Figure 2. A total of 82 participants (fifth year: n=77, 94% and sixth year: n=5, 6%) agreed to answer the surveys (response rate 82/112, 73%).

**Table 2:** Survey results for questions 1-4, 11, and 12 (N=82, response rate 82/112, 73%).

Question implication		Responses
**Q1. Satisfaction**		
	Excellent (5), n (%)	23 (28)
	Very good (4), n (%)	37 (45)
	Good (3), n (%)	18 (22)
	Fair (2), n (%)	4 (5)
	Poor (1), n (%)	0
	Mean (SD)	3.94 (0.83)
**Q2. Advantages (multiple options allowed, n=159), n (%)**		
	I could participate while seated	42 (26)
	I could closely observe the examination	41 (26)
	I could listen to the faculty’s commentary	39 (25)
	It was easy to understand because the points to focus on were shown	34 (21)
	I did need not care about not being in the way	1 (1)
	Nothing	2 (1)
**Q3. Drawbacks (multiple answers allowed, some options already prepared, n=80), n (%)**		
	Lack of an immersive experience	40 (50)
	Poor video and audio quality	19 (24)
	Lack of a sense of tension	7 (9)
	I could not look at the fine movements of the patient	1 (1)
	I could not look at the patient’s expression from my preferred perspective	1 (1)
	Not better than in-person participation	1 (1)
	Nothing	11 (14)
**Q4. Self-rated learning value, n (%)**		
	Video ward rounds are better	26 (32)
	In-person rounds are better	21 (25)
	Both are equal	35 (43)
**Q11. Modeling professionalism, n (%)**		
	Felt in video ward round	10 (12)
	Felt in both	48 (59)
	Felt in in-person round	19 (23)
	Could not feel in either	5 (6)
**Q12. Change in motivation, n (%)**		
	Influenced	53 (65)^a^
	Unchanged	29 (35)

^a^*P*=.004 in *z* test.

**Table 3:** Survey results for questions 5-10 (N=82).

Observation of abnormal neurological findings	Could observe in video ward round, n (%)	Could observe in both, n (%)	Could observe in in-person round, n (%)	Could not observe in either, n (%)	Significance in adding video ward round to in-person round (*P* value)	Effect size (Cohen *h*)
Q5. Babinski sign (extensor plantar response)	18 (22)	20 (24)	12 (15)	32 (39)	.34	0.11
Q6. Hyperreflexia (exaggerated response to tendon tap)	18 (22)	24 (29)	16 (20)	24 (29)	.76	0.03
Q7. Cerebellar ataxia on the finger-to-nose test	13 (16)	20 (24)	13 (16)	36 (44)	≥.99	0.21
Q8. Involuntary movement (eg, tremor)	19 (23)	14 (17)	10 (12)	39 (47)	.14	0.09
Q9. Muscular weakness	17 (21)	34 (42)	12 (15)	19 (23)	.42	0.20
Q10. Abnormal sensory examination	24 (29)	25 (30)	10 (12)	23 (28)	.01	0.38

### Program Strengths and Weaknesses, Satisfaction, and Self-Rated Learning Value

In question 1, which focused on overall satisfaction, the mean score of participants’ satisfaction for the remote round was 3.94 (SD 0.83; excellent 5 and poor 1). No participant scoring 1 was noted.

Common responses to question 2, regarding the strengths of remote rounds, included “I could closely observe the examination,” “It was easy to understand because it showed the points to focus on,” “I could listen to the faculty’s commentary,” and “I could participate while seated.” On the other hand, most of the responses related to the weaknesses were that there was a lack of immersion compared to face-to-face interaction. Regarding the comparison to in-person rounds, we asked the students to rate the learning effects of remote rounds if they involved the same amount of learning time as in-person rounds. All students experienced attending in-person and remote rounds and 43% (35/82) felt that both learning effects were equal, while 32% (26/82) of the participants preferred remote rounds to in-person rounds.

### Additional Experiences

One of the important learning objectives of participation in neurology ward rounds is seeing abnormal examination findings in real patients. In questions 5 to 10, we asked whether the student observed major abnormal neurological findings (Babinski sign, hyperreflexia, cerebellar ataxia, involuntary movement, muscular weakness, and abnormal sensory examination). Each student observed abnormal findings from assigned patients during in-person examinations, both by themselves and watching examinations performed by the professor; however, students never saw findings from patients assigned to other students. In addition to in-person rounds, remote ward rounds increased the number of students who witnessed abnormal neurological findings by a mean of 22% (SD 3.5; Figure 2 and Table 3). Although involuntary movements such as tremors were rarely observed in ward rounds, adding live video streaming with in-person rounds resulted in at least 53% (43/82) of students having the opportunity to gain experience. Adding video ward rounds to in-person rounds in sensory testing statistically significantly increased the chance of watching neurological findings (Q10, *P*=.01).

### Changes in Motivation and Behavior

Regarding professionalism, we asked, “In which round format did you feel that the professors and supervisors were more attentive and caring toward the patients?” Most of the participants answered that they felt these attitudes in both rounds, but few answered remote rounds only. However, in the final question, “Did your neurology rotation training, including remote participation in the professor’s rounds, influence your motivation during your subsequent clinical clerkship?” In total, 65% (53/82) of the participants replied “influenced” with statistically significant (*P*=.004).

## Discussion

### Principal Findings

The on-premises web conferencing system enabled live-streaming of the professor’s neurology rounds in a separate room in the hospital without concern regarding patients’ personal information being compromised. Participation in remote rounds could enhance learning experiences in undergraduate neurology rotators before entering a genuine clinical clerkship, especially through the observation of abnormal neurological findings such as tremors.

As the COVID-19 pandemic has resulted in inadequate undergraduate clinical education, various online education initiatives have been undertaken at teaching centers worldwide. However, owing to the lack of extensive experience with educational initiatives involving live streaming of medical examinations before the pandemic, an adequate online educational milieu could not be prepared within a short time [8]. The underdevelopment of this initiative may be attributed to the fact that before the COVID-19 pandemic, there was no need to broadcast examinations to other locations since there were no restrictions on student contact with patients. Another reason may have been the belief among educators that learning directly at the patient’s bedside was the best method for medical students. There is no doubt that much can be learned through observing patients directly. Interestingly, previous studies indicated that in-person teaching formats with patient contact were rated significantly higher by students compared to non–in-person teaching, but that there was no particular difference in students’ knowledge and skill development compared to non–in-person learning [9]. Another previous study showed that there is no difference between the effectiveness of teaching a procedure by conventionally demonstrating it in the field and via prerecorded videos [10]. On-demand video education, which can be repeated in one’s own time, is considered to be the favorable method for routine skill training for well-established procedures that do not vary from case to case. On the other hand, remote live learning sessions, which include virtual bedside teaching rounds, were carried out at the height of the pandemic because direct contact with the patient was to be avoided [11]. In-person learning at the bedside is a better teaching method than any textbook for the management of acute illnesses, as findings vary for each case and conditions can change on an hourly basis during the same disease. In addition, the findings of neurological examinations in neurological diseases do not always manifest with the same pattern (eg, a lesion in the semilateral cervical spinal cord does not always indicate Brown-Sequard syndrome), and experience with a large number of cases can make the neurological examination a useful tool even in the current neuroimaging-driven milieu [12].

There is a tacit agreement that the medical institution will never divulge an individual’s private information, which forms the basis of the relationship of trust between the medical institution and patients in Japan. Therefore, extreme reassurance that “the hospital information network must not be connected to the internet” was commonly required. Hofmann et al [11] used HIPAA-compliant Zoom to live-stream COVID-19 rounds, which required financial investment. Now that the pandemic has subsided, the additional cost of this system should be balanced with the benefits. There is a tendency to cut costs in medical education, where the rewards are difficult to see. Moreover, it is easy to regress to the mindset of shying away from new, high-risk educational initiatives rather than incurring high costs to mitigate the security risks of leaking patients’ personal information. Countries such as Australia, which have largely adopted the common use of web conferencing software in clinical practice, are rare [13]. Tremendous effort is required to obtain the consent of the facility manager from the perspective of network security and personal data protection in our country. Therefore, the implementation of hospital-based apps using open-source technology without additional cost, as with our initiative, can be achieved easily and be useful if the hospital’s information technology department is staffed by technically competent personnel.

Undergraduate medical education in Japan is based on the “Model Core Curriculum for Medical Education in Japan,” which is a systematically organized model that is formed by extracting the core parts of the curriculum that should be commonly addressed by all Japanese universities when formulating their own medical education curricula. [14]. In this curriculum, the learning objectives are defined in the “learning and assessment items related to skills and attitudes required at the beginning of clinical clerkship” reported by the public interest incorporated association, Common Achievement Tests Organization. Some neurological signs and how to perform a neurological examination were described in the textbook. However, since practice before Objective Structured Clinical Examinations is based on examining normal patients, one of the learning objectives of clinical rotations in neurology is to observe genuine pathological neurological findings in the patients. Although various pathological findings should be shown at clinical rounds for all students as often as possible, those chances were extremely limited because of the short duration of bedside teaching in the curriculum and because satisfactory education needs additional effort from the tutor [15]. The experience of examining assigned patients and observing abnormal findings, such as hyperreflexia and the Babinski sign, was expected to motivate the learners, but the lack of abnormal findings in assigned patients biased their experience. Remote participation in the professor’s rounds could overcome their inequality and drive learning motivation. Indeed, our initiative led to 22% (18/82) more students having opportunities to see abnormal neurological signs compared to conventional teaching, without any additional tutor effort. During their 6 years in medical school, all students underwent only 5 days (40 h) of bedside education in neurology, of which less than 1 hour was spent attending the rounds conducted by professors, unless they selected neurology as their elective practice in the final year. For those with little clinical experience, the “memory” of the experience is more important than the acquisition of skills at this stage and 65% (53/82) of participants answered that neurology rotation including remote rounds influenced learning motivation during subsequent rotations. Attending a professor’s rounds, such as “Charcot's Tuesday lectures,” is impressive and informative even for brief periods and should be essential for undergraduate education in neurology, even if conducted remotely [16]. Our initiative can not only add educational value to conventional education methods but also help important traditional aspects of neurology education to continue, using novel technologies.

### Limitations

A key limitation of this study is that not every medical education facility has the staff to build an in-house web conferencing system such as KUMEX that can be successfully adapted to the facility’s system. When they try to outsource the installation of a similar system, they are often hesitant to implement it because of the high initial and maintenance costs. The fact that most hospitals and medical education facilities do not have dedicated information technology specialists on staff may also be relevant.

Second, in verifying the effectiveness of this initiative precisely, a comparison should originally have been made between 2 groups, an intervention group and a control group. Since it is difficult to modify the curriculum for research purposes, our initiative was designed as an addition to the current curriculum (in which all participants experience in-person rounds, even if only for a short time), resulting in a comparison of impressions from each individual, which may have left a more favorable impression of the remote rounds with their longer time and explanations.

Third, the effectiveness of the remote rounds was not evaluated by objective tests, but only by calculating the number of students who observed abnormal findings. Evaluating the true learning effects of conducting remote rounds requires a permanent introduction of remote rounds and a comparison of the level of academic achievement between the grades before and after the introduction of remote rounds, and it is expected that a larger-scale survey will be conducted based on this pilot study.

Finally, no active research has been conducted on the psychological safety of patients. None of the personal information was leaked, but it is possible that patients may have felt uncomfortable. Future studies should investigate the feelings on the part of patients who feature in the relayed scenes of medical examinations.

### Conclusions

Live-streaming of neurology ward rounds using a secure in-house web conferencing system can be achieved easily and is useful for undergraduate education without concerns about patients’ personal information being compromised. This initiative could enhance learning by providing direct experience of observing neurological findings before entering clinical clerkship and driving learning motivation. Furthermore, live-streaming of rounds may be a novel style of education that is more effective than the conventional education process of attending tutor rounds with some constraints.

## Abbreviations

HIPAA: Health Insurance Portability and Accountability Act
KUMEX: Kobe University School of Medicine Web Conference Lecture System
VLAN: virtual local area network
WebRTC: Web Real-Time Communication

## Data Availability

Data from this study are available upon reasonable request from the corresponding author.
